# Xuesanqi ameliorates DSS-induced colitis in mice by mediating gut microbiota dysbiosis and modulating MAPK/ERK/JNK pathway

**DOI:** 10.1007/s13659-024-00482-8

**Published:** 2024-12-01

**Authors:** Qiyuan Su, Qian Hu, Songtao Wu, Suqin Yang, Hanwen Su, Zhengjun Zhang, Chengxiu Ling

**Affiliations:** 1 Wisdom Lake Academy of Pharmacy, Xi’an Jiaotong-Liverpool University, Suzhou, 215123 Jiangsu People’s Republic of China; 2 Wuhan Children’s Hospital, Tongji Medical College, Huazhong University of Science and Technology, Wuhan, 430016 Hubei People’s Republic of China; 3 Faculty of Pharmacy, Hubei University of Chinese Medicine, No. 16, Huangjiahu West Road, Hongshan District, Wuhan, 430065 Hubei People’s Republic of China; 4 School of Pharmaceutical Sciences, South-Central Minzu University, Wuhan, 430074 Hubei People’s Republic of China; 5 Department of Clinical Laboratory, Renmin Hospital of Wuhan University, Wuhan, 430060 Hubei People’s Republic of China; 6 Department of Statistics, University of Wisconsin-Madison, Madison, WI 53706-1481 USA; 7 School of Economics and Management, and MOE Social Science Laboratory of Digital Economic Forecasts and Policy Simulation, University of Chinese Academy of Sciences, Center for Forecasting Sciences, Chinese Academy of Sciences, Beijing, 100049 People’s Republic of China

**Keywords:** Colitis, Xuesanqi, Gut microbiota, Short chain fatty acids (SCFas), MAPK/ERK/JNK signaling pathway, Tight junction (T.J.) protein

## Abstract

This study aimed to evaluate the therapeutic properties of the traditional Chinese medicine Xuesanqi (XSQ, from the rhizome of *Polygonum amplexicaule* D. Don) in treating ulcerative colitis. We hypothesized that its many active components can alleviate symptoms of colitis by regulating the gut microbiota, its metabolites, and various signaling pathways. To test our hypotheses, we designed a DSS- induced colitis model in C57BL/6 male mice. Apparent metrics were evaluated in each group of mice and performed histological analysis of relevant tissues. The gut microbial composition was analyzed by 16S rRNA sequencing of bacteria. Simultaneously, the SCFAs content was detected by gas chromatography, inflammatory factor secretion was evaluated by ELISA or western-blot, the expression of tight junction protein and key proteins of the MAPK signaling pathway were analyzed by western-blot. Our result showed that the treatment with XSQ alleviated significant various symptoms such as weight loss, blood in stool, and shortening of colon. In addition, XSQ treatment restored the dysregulated gut microbiota in colitis mice, increased short chain fatty acids (SCFAs) and normalized the MAPK/ERK/JNK signaling pathways, promoted expression of tight junction protein Occludin, Claudin-1, and E-cadherin proteins. Furthermore, we also observed a dose-dependent pattern in these treatment responses. These findings demonstrated the active components of XSQ is a promising new treatment platform for ulcerative colitis.

**Graphical Abstract:**

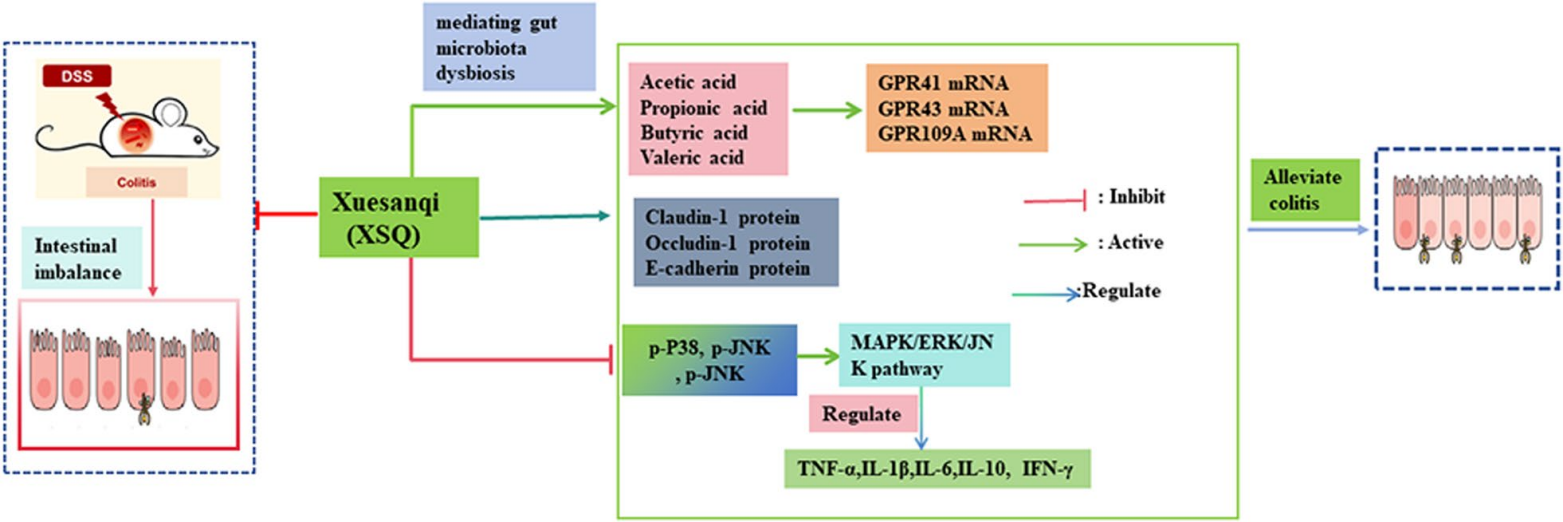

## Introduction

Colitis is a persistent and sometimes refractory inflammatory bowel disease (IBD) with untold etiology [1]. Current literature indicates environmental exposure, luminal factors, genetic heredity, and immune response as possible causes [1–5]. Symptoms of colitis include diarrhea, abdominal pain, and blood in stool. These quality-of-life-altering symptoms made rapid remission a priority in colitis treatment. Conventional treatment of colitis is the administration of 5-aminosalicylic acid drugs or corticosteroid injections in more advanced cases [5]. However, in many cases, these treatments have failed to prevent relapse or suppress negative symptoms altogether [6]. Colitis significantly elevates patients’ risk of developing serious complications and colorectal cancer, while there is a lack of effective treatment with miniature side effects [7].

Lately, gut microbiota has been a focal point of colitis studies. Current literature reports that colitis patients’ gut microbiota has impaired diversity and balance, and the dysregulated gut microbiota may play a significant role in colitis’s manifestation. Veltkamp et al. found that a sterile environment prevents intestinal inflammation in genetically susceptible mice; conversely, implanting pathogenic bacteria or intestinal microbiota from colitis patients into healthy mice induced inflammation and leads to a strong immune response that exacerbates the inflammation [8]. Another study argued that probiotics found in breast milk, such as *Lactococcus* and *Bifidobacterium*, may help prevent colitis by regulating the gut microbiota and its metabolites as well as maintaining the integrity of the intestinal barrier [9]. In addition, short-chain fatty acids (SCFAs), metabolites from the gut microbiota, are thought to improve autoimmunity. Thus, modulating the imbalance of the gut microbiota and increasing the levels of SCFAs may play an important role in the treatment of colitis.

The root of the rhizome of *Polygonum amplexicaule* D. Don, locally known as Xuesanqi (XSQ), is a widely used herbal medicine in Enshi Ethnic Minority Area, Hubei Province, China. Traditional Chinese Medicine (TCM) texts recorded that XSQ has the properties of clearing away heat and detoxifying, activating blood circulation and removing blood stasis, and is traditionally used for treatment of gastroenteritis and dysentery [10]. Modern pharmacological studies have shown that XSQ has antibacterial, anti-inflammatory, and anti-atherosclerotic effects, but its therapeutic studies in colitis have not been reported, while the clinical symptoms of colitis are similar to those of diarrhea and gastroenteritis. Therefore, we hypothesize that XSQ can alleviate colitis’s pathological symptoms by regulating intestinal flora imbalance and increasing the content of SCFAs, which are metabolites of flora. Moreover, SCFAs can inhibit the activation of intracellular inflammatory signaling pathway by activating cell membrane surface G-protein-coupled receptors, thus inhibiting the secretion of inflammatory factors suppressing the inflammatory response, and promoting the repair of intestinal homeostasis; moreover, the recovery of colitis is related to the expression of tight junction proteins, so can XSQ promote the expression of tight junction proteins? It is worth exploring.

To verify our hypothesis, we designed a mouse clinical trial that featured DSS-induced mice colitis models. The main outcome of interest is measurable colitis-related symptoms, such as changes in gut microbial composition, SCFA levels, inflammatory signaling pathways, and more. In this study, our result propounded that XSQ can induce remission of symptoms in colitis mice, including decreased disease activity index (DAI), and longer colon length in colitis mice. Besides the above metrics, XSQ restored the imbalance in gut microbiota populations, and increased metabolism of SCFAs, especially acetic acid and propionic acid. We further observed that XSQ can upregulate the expression of tight junction proteins, such as Occludin, Claudin-1*,* and E-cadherin, and improve the integrity of the intestinal barrier. Additionally, XSQ can relieve the inflammatory response by regulating the secretion of inflammatory cytokines IL-6, IL-1β, anti-inflammatory cytokine IL-10, and so on. These discoveries support our hypothesis that XSQ promotes the recovery of colitis. XSQ may offer a superior alternative to current colitis treatment in the future. Our research has highlighted the remarkable potential of repurposing TCM, while also establishing a robust framework for future translational medicine research.

## Result

### UPLC-Q-TOF–MS/MS analysis of XYKJP components

**Fig. 1:**
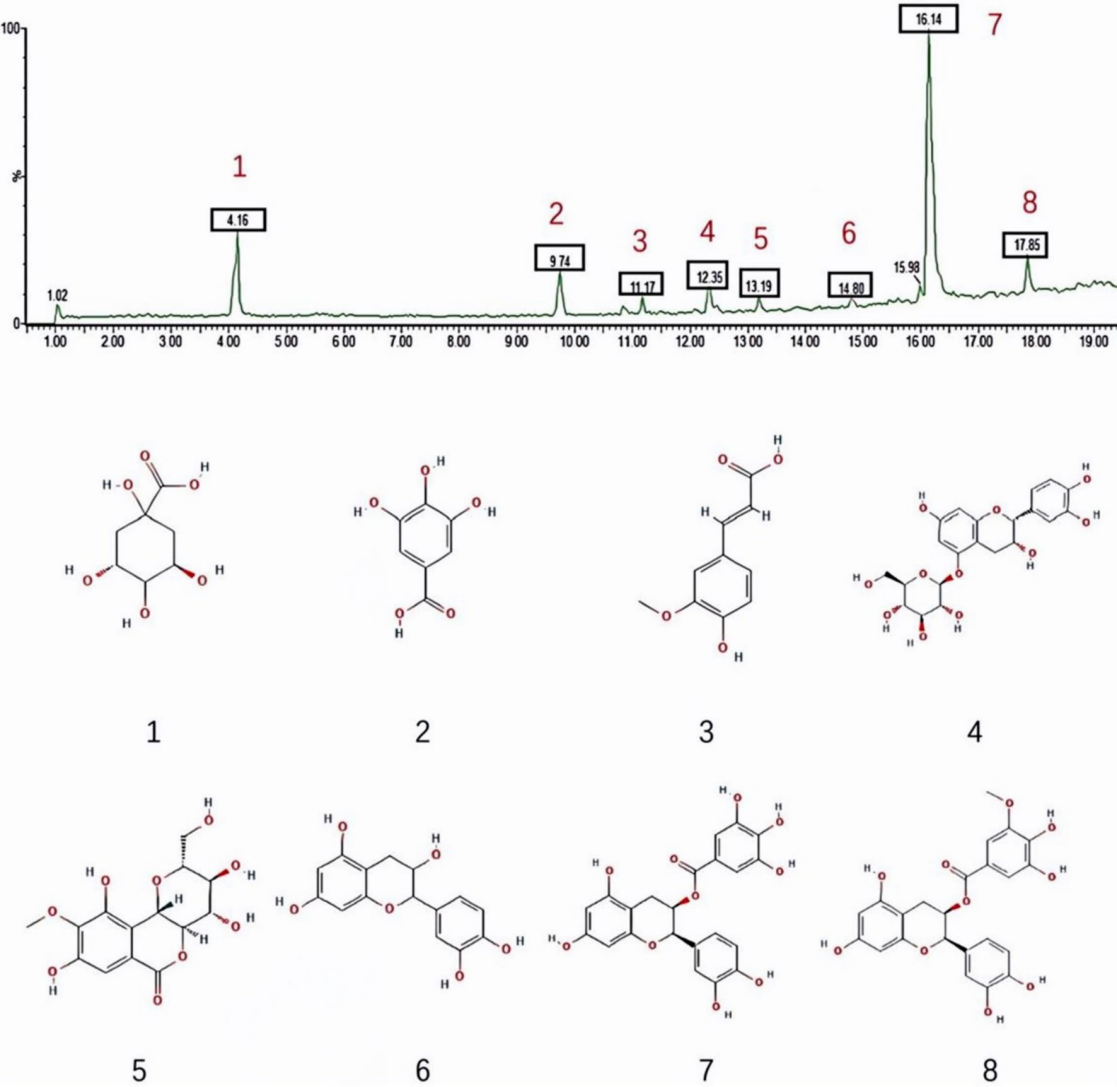
Analysis of active components in XSQ by UPLC-Q-TOF–MS/MS. Peak 1: Quinic acid; Peak 2: Gallic acid; Peak 3: Ferulic acid; Peak 4: (+)-catechin-5-O-glcolitisoside; Peak 5: Rock cabbage; Peak 6: Epicatechins; Peak 7: Epicatechin gallate Feruloylquinic acid; Peak 8: Epicatechin-3-O-(3-O-methyl) gallate

To elucidate the chemical profile of the XSQ extract, a comprehensive UPLC-Q-TOF–MS/MS analysis was performed following a stringent chromatographic cleanup process. The analysis of these components showed that they have a certain content, which indicates that they may be the material basis for pharmacological activity. The precise concentrations of quinic acid (4.02 mg/g), gallic acid (1.93 mg/g), ferulic acid (0.62 mg/g), (+)-catechin-5-O-glucoside (0.33 mg/g), rock cabbage (0.59 mg/g), epicatechins (0.81 mg/g), epicatechin gallate feruloylquinic acid (10.81 mg/g), epicatechin-3-O-(3-O-methyl) gallate (1.54 mg/g) underscore its importance as key biomarkers for the quality assessment of the botanical material in question (Fig. 1).

### Response to treatment and dose–response study

**Fig. 2:**
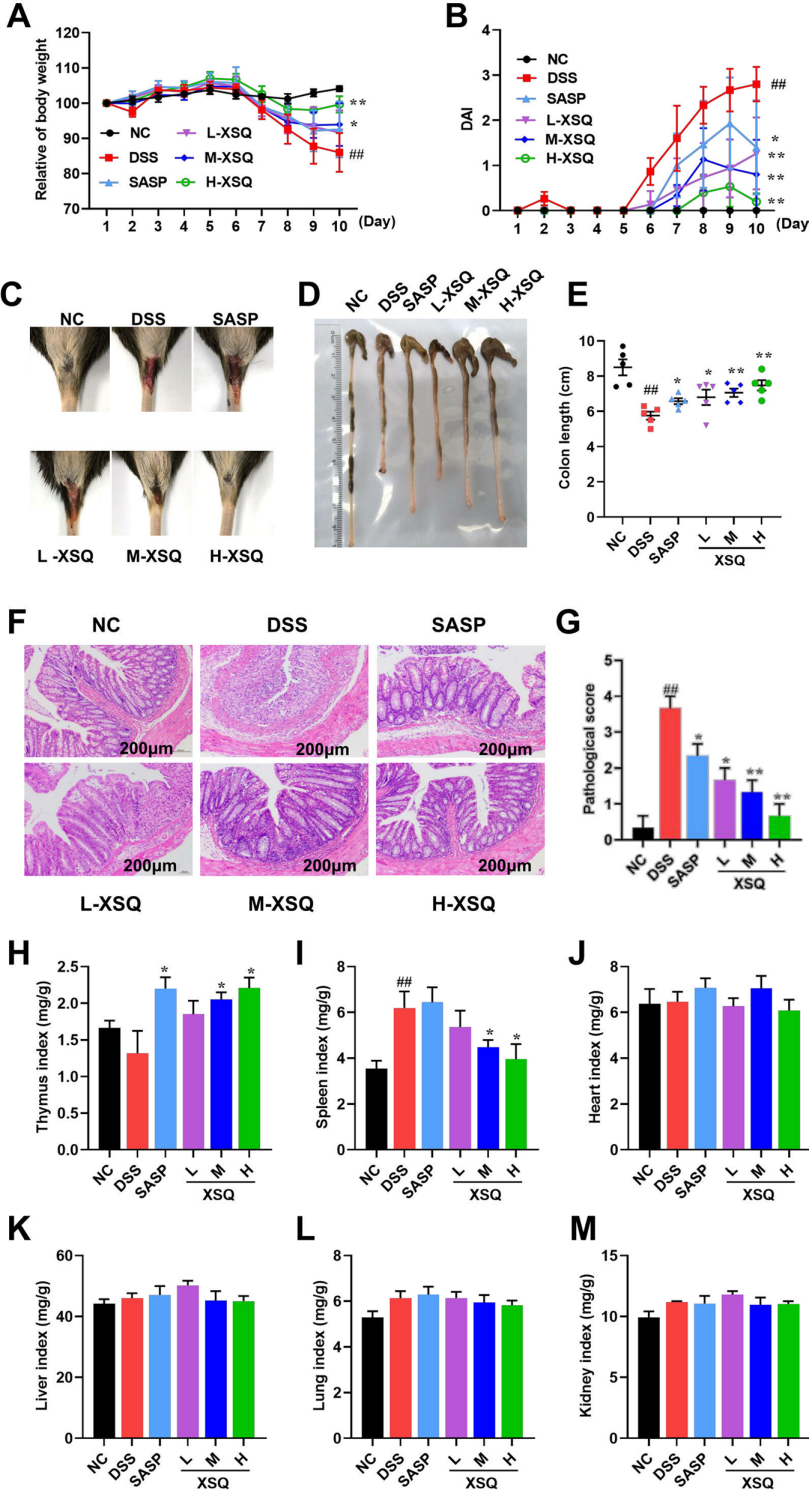
XSQ alleviates DSS-induced colitis symptoms and is safe. **A** Each group’s mice body weight changed daily within ten days. **B** The result of the DAI score was estimated daily. **C** The result of representative stool observations for each group of mice. **D**, **E** The result of each group’s mice colon length was measured and then calculated. **F**, **G** The result of representative H&E images of the colon (scale bars = 200 μm) were captured to obtain the pathological evaluation. **H-M** The thymus, spleen, heart, liver, lung, and kidney indices were calculated. Data were expressed as ± SEM (n = 10), *P < 0.05, **P < 0.01, ***P < 0.001

The potential of XSQ to alleviate colitis symptoms was explored*.* As Fig. 2A, B shows, the weight in the DSS group had a significant decrease, and the DAI scores had a significant increase compared to the Ctrl group, which was significantly reversed in the XSQ treatment group in a clear dose-dependent difference (*P* < 0.05). In addition, as illustrated in Fig. 2C, there was a clear dose-dependent difference in blood in the stool, with mice treated at higher doses tending to have less severe symptoms.

When there is inflammation or ulceration in the colon tissue, the colon compensatorily contracts and becomes shorter. The severity of inflammation and ulceration may be reflected in the degree of colon shortening. We observed statistically significant shortening of the colon in the DSS group. Conversely, the shortening of the colon is significantly less severe in the XSQ group with dose-dependent differences (Fig. 2D, E). In addition, histological analysis revealed severe inflammatory cell infiltration in the colon as well as evident lesions of goblet cells in the DSS group; however, XSQ could significantly alleviate colon damage and inflammatory infiltrate (Fig. 2F, G). These observations, in combination, showed that XSQ treatment can improve symptoms in DSS-induced colitis, and there is a dose-dependent response in the degree of remission.

At the same time, we evaluated relevant organ indices to assess the safety of XSQ administration. As was shown in Fig. 2H, I, XSQ increases the thymus index while decreasing the spleen index. Moreover, XSQ does not affect heart, liver, lung and kidney indices. The above results indicate that XSQ is not only effective for colitis but also is safe.

### XSQ modulates cytokine secretion in DSS-induced colitis mice

**Fig. 3:**
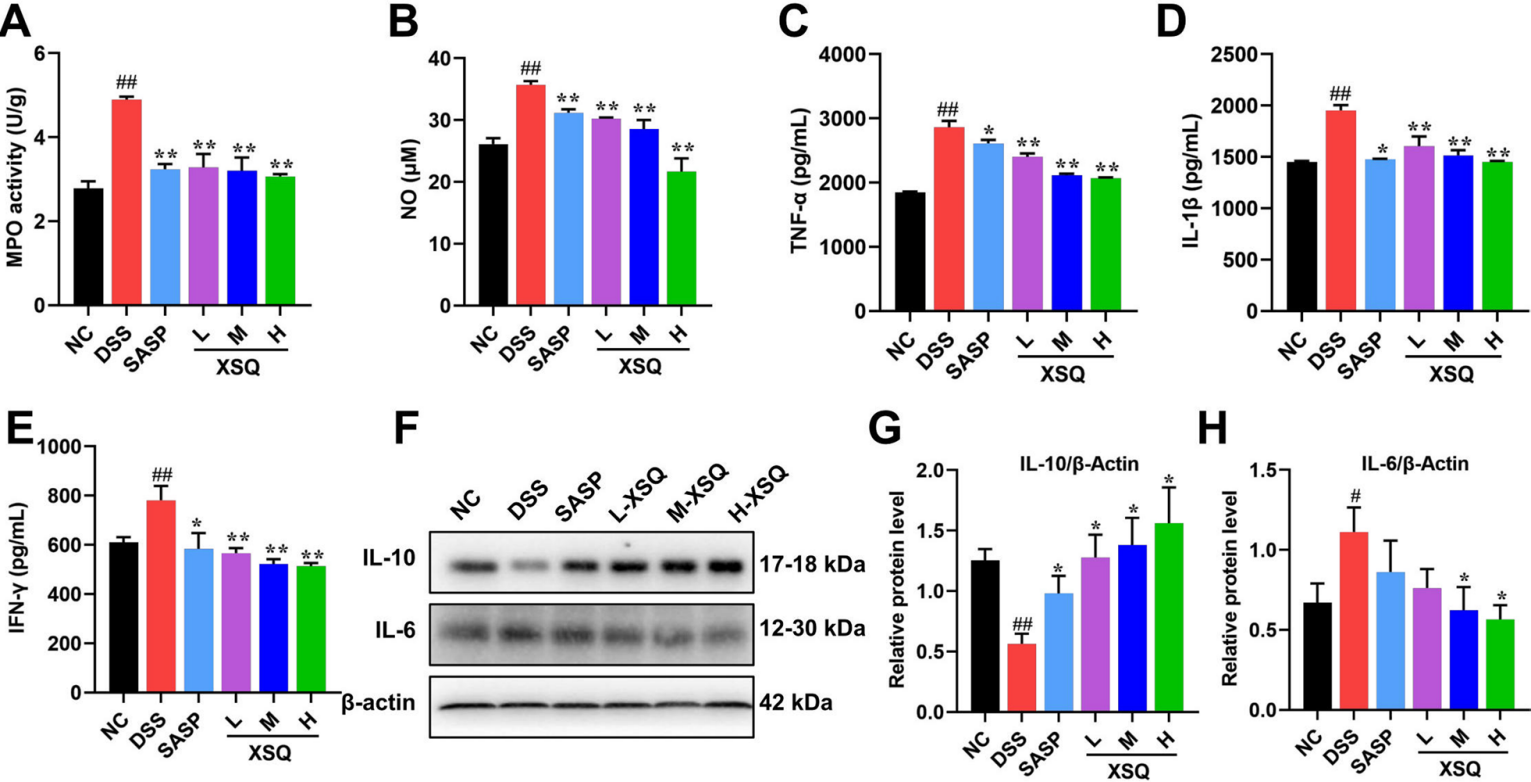
XSQ regulates the inflammatory cytokine expression in DSS-induced colitis mice. **A**, **B** The result of the MPO and NO activity of colon tissues; **C**–**E** The ELISA statistical results of TNF-α, IL-1β, and IFN-γ. **F** The representative images of western blotting of IL-6 and IL-10. **F**–**H** The expressional changes statistical results of IL-6 /β-Actin and IL-10 /β-Actin protein by Image J software. Data were expressed as ± SEM (n = 3), *P < 0.05, **P < 0.01, ***P < 0.001

Dysregulated cytokines are strongly associated with the manifestation of colitis. To explain this association, the inflammatory mediators NO and MPO were examined first, and it was found that XSQ significantly reduced their levels (Fig. 3A, B). Next, we used the ELISA and the western blot methods to access the various cytokines in the colon tissues. The comparison of various pathways, including TNF-α, IL-1β, IFN-γ, IL-6, and IL-10 is visualized respectively in Fig. 3. In the DSS group, the results showed a significant increase in TNF-α, IL-1β, and IFN-γ, respectively. However, XSQ can suppress their secretion dose-dependently (Fig. 3C–E). In addition, we assessed the expression of inflammatory factors of IL-6 and IL-10 using western blot to confirm the anti-inflammatory effects of XSQ further. The results showed a significant decrease in IL-10 protein expression and an increase in IL-6 protein expression in the DSS group. In contrast, XSQ can significantly reverse these inflammatory factors’ imbalance outcomes in a dose-dependent manner, indicating that XSQ treatment effectively suppresses the inflammatory reactions of DSS-induced colitis (Fig. 3F–H,* P* < 0.05 or *P* < 0.01). Therefore, based on these results, the anti-inflammatory efficacy of XSQ is exciting.

### Result of 16s rRNA sequencing

**Fig. 4:**
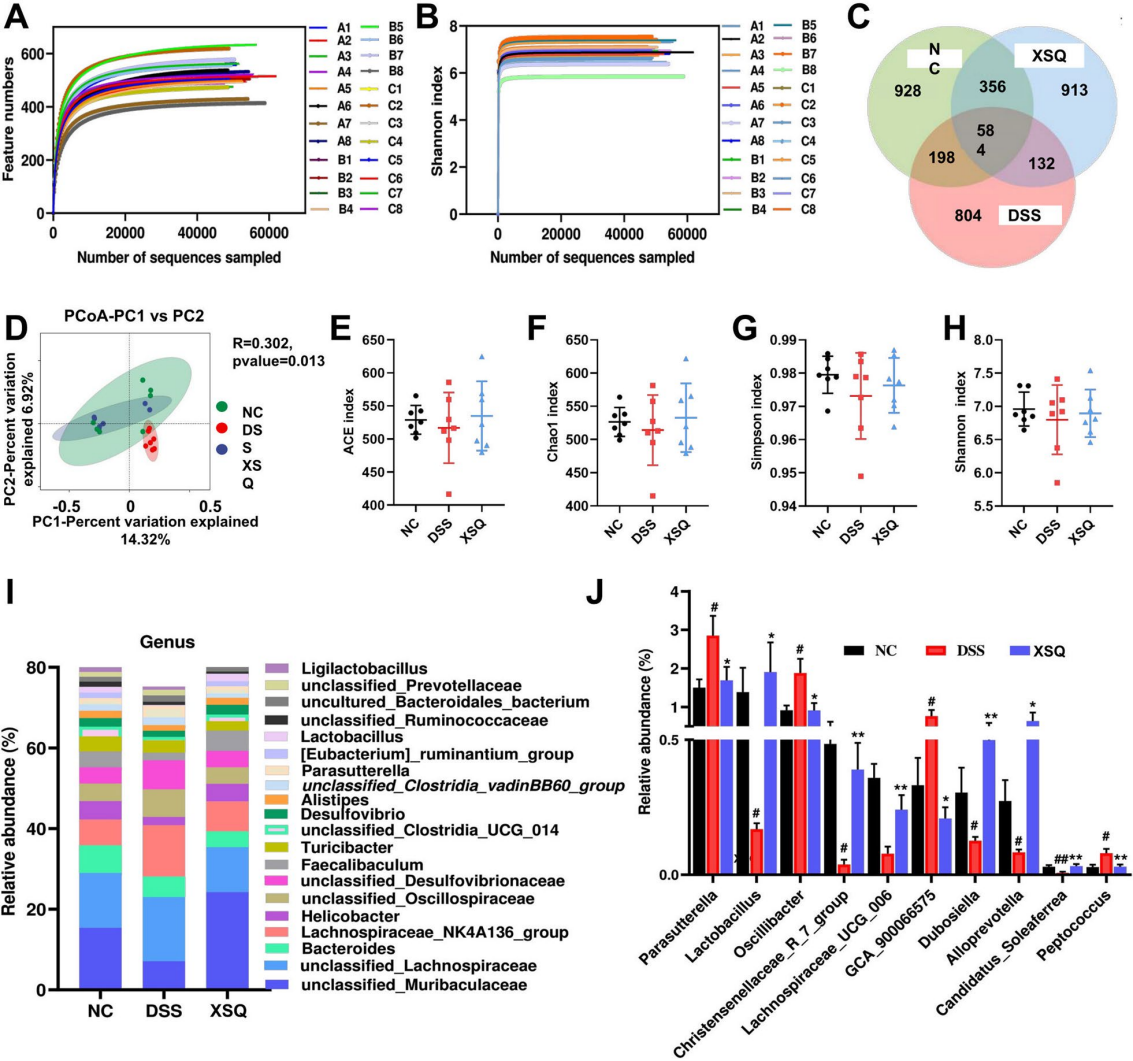
SXQ regulates gut microbiota imbalance DSS-induced colitis in mice. **A** Dilution Curve; **B** Shannon’s index curve; **C** Venn diagrams; **D** The figure of PCoA based on binary_jaccard distance algorithm. **E** ACE index; **F** Chao index; **G** Simpson index; **H** Shannon index; **I** Changes in the relative abundance of intestinal flora at the genus level; **J** Differential bacterial abundance changes at the genus level. Data were expressed as ± SEM (n = 7), ^##^*P* < 0.01 vs Ctrl group; *P < 0.05, **P < 0.01 vs DSS group

The 16 s rRNA sequencing results yielded 1,657,766 high-quality tag sequences. After visualization, we observed parallel smooth curves with shallow slopes in the Shannon index plot. This result indicates that, between different groups, there are differences in species richness but a uniform distribution of species evenness (Fig. 4A, B). We plotted Venn diagrams to show results for operational taxonomic units (OTUs). The Ctrl group, DSS group, and XSQ group had 928, 804, and 913 unique OTUs, respectively, and shared 584 common OTUs (Fig. 4C). Next, we compared the α-diversity and β-diversity of gut microbiota between groups using QIIME 2™ (National Institutes of Health, U.S.) [11]. We found that the α- diversity in the XSQ group increased significantly in response to treatment, while β diversity remained unchanged (Fig. 4E–H). Principal Coordinates Analysis (PCoA) suggested that DSS-induced colitis significantly altered the diversity of bacterium in the DSS group, while the XSQ group and the Ctrl group had less difference. These results suggest that XSQ treatment can regulate the gut microbiota imbalance in DSS-induced colitis mice (Fig. 4D).

### XSQ regulates gut microbiota in DSS-induced colitis mice

**Fig. 5:**
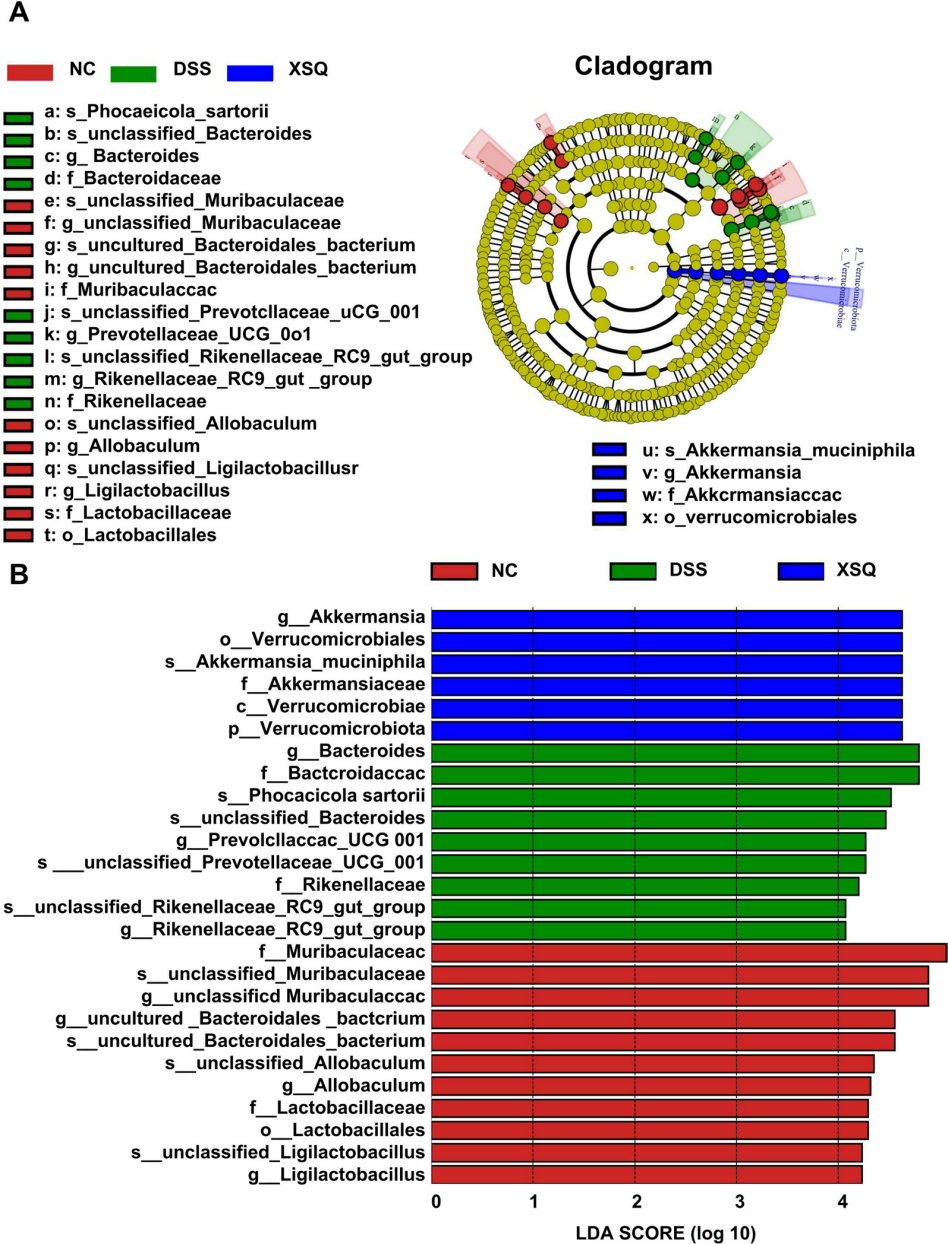
SXQ regulates gut microbiota imbalance DSS-induced colitis in mice. **A** Linear discriminant analysis (LDA) of taxa in three experimental groups; **B** Linear Discriminant Analysis (LDA) Scores

We carried out quantitative studies of species abundance to identify species with significant changes in abundance. Compared to the Ctrl group, the DSS group significantly decreased in abundance in 16 genera, including *Helicobacter*, *Candidatus Saccharismonas*, *Lactobacillus*, *Dubosiella*, *Alloprevotella*, *Lachnospiraceae COLITISG-006*, and more. Simultaneously, a significant increase in abundance in 16 genera, including *Lachnospiraceae NK4A136-group*, *unclassified Desulfovibrionaceae*, *Parasutterella*, *Oscillibacter*, *GCA-900066575*, *Blautia*, *Acinetobacter*, and more. Compared to the DSS group, the XSQ group significantly decreased in 18 genera, including *unclassified Oscillospiraceae*, *Parasutterella*, *Oscillibacter*, *Odoribacter*, *unclassified Peptococcaceae*, *Bilophila*, and more. Simultaneously, significant increase in 30 genera, including *unclassified Muribaculaceae*, *Lactobacillus*, *Eubacterium Ruminatium Group*, *Parabacteroides*, *Alloprevotella*, *Dubosiella*, and more. Overall, XSQ treatment can reverse 37.5% and 37.5% of genera increase and decrease in species abundance, respectively, indicating that XSQ can help regulate the imbalance in gut microbiota caused by DSS-induced colitis. We used a linear discriminant analysis effect size approach (LDA) to identify general changes associated with each group. We identified 6 genera associated with the XSQ group, 9 with the DSS group, and 11 with the Ctrl group using a score of more than 4 as a threshold [12] (Fig. 5A, B).

### XSQ promoted the production of short-chain fatty acids and activated the G-protein-coupled receptors

**Fig. 6:**
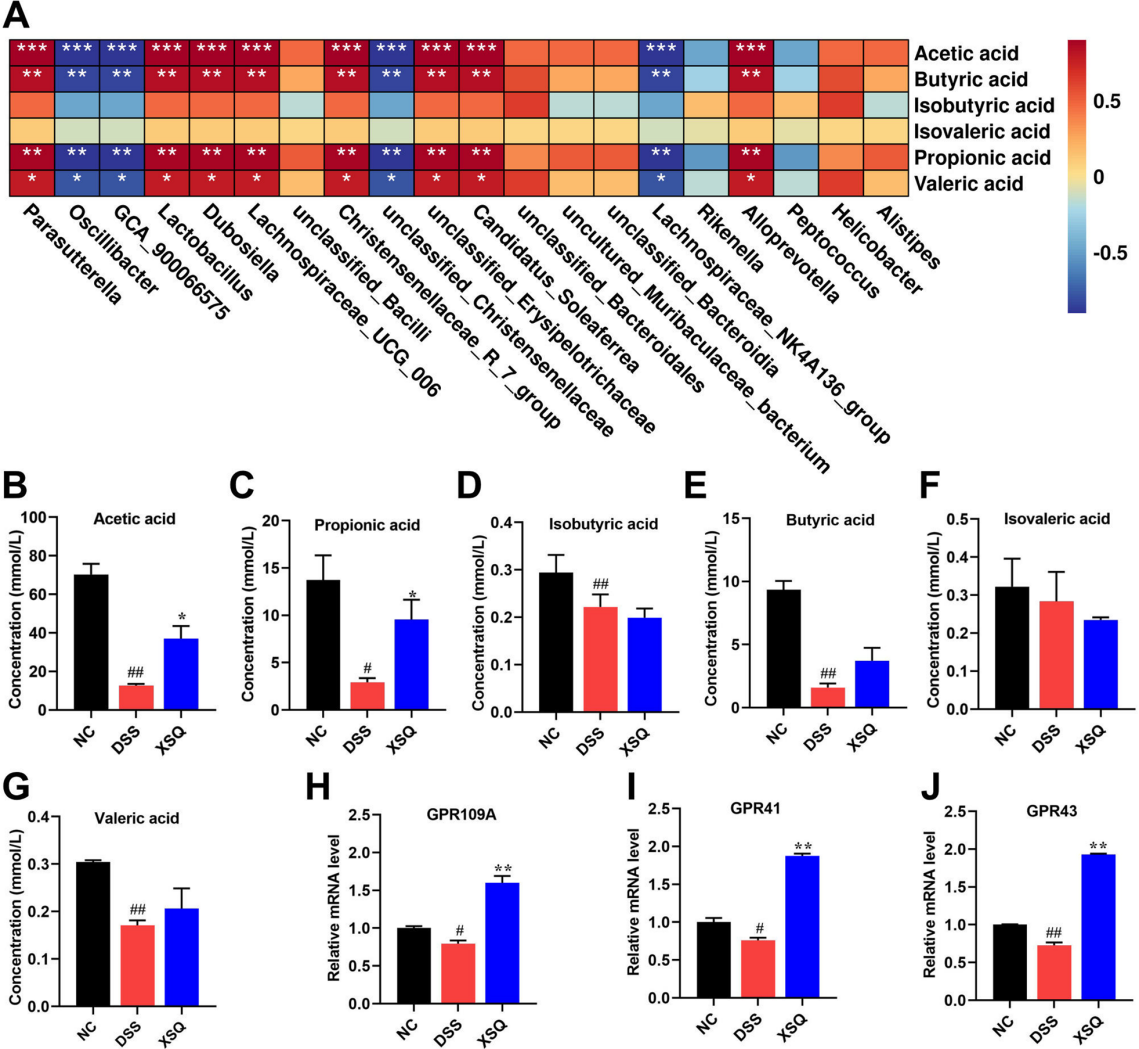
XSQ promoted the secretion of SCFAs and the expression of the GPR41/43/109A gene. **A** Heatmap analysis of the correlation between differential Bacteria and SCFAs; **B** Acetic acid content; **C** Propionic acid content; **D** Isobutyric acid content; **E** Butyric acid content; **F** Isovaleric acid content; **G** Valeric acid content; **H** The mRNA level of GPR41; **I** The mRNA level of GPR43; **J** The mRNA level of GPR109A. Data were expressed as ± SEM (n = 3), ^##^*P* < 0.01 vs DSS group; *P < 0.05, **P < 0.01 vs Ctrl group

Balanced secretion of SCFAs is a pivotal indicator of the heterogeneous relationship between the host and the gut microbiota. SCFAs are one of the metabolites of intestinal flora, and the above results show that XSQ promotes the abundance of short-chain fatty acid-producing beneficial bacteria (Fig. 6A). We used the G.C. method to measure the secretion of SCFAs in the cecum. As Fig. 6B–G shows, we observed a significant decrease in the levels of acetic acid, propionic acid, butyric acid, and valeric acid compared to the Ctrl group in the DSS group. However, XSQ markedly increased the levels of acetic acid (Fig. 6B) and propionic acid (Fig. 6C), at the same time increasing the trend in butyric and valeric acid content in the XSQ group (Fig. 6E, G) while the content of isovaleric acid was slightly reduced in the XSQ group (Fig. 6F). These results indicate that XSQ treatment restores the dysregulation of SCFAs levels in DSS-induced colitis.

SCFAs can activate G-protein receptors, such as GPR41/43/109A, on the membrane of intestinal epithelial cells, thus inducing a series of physiological activities in the intestinal epithelial cells, such as inhibition of the inflammatory response. Thus, the mRNA of GPR41/43/109A was detected. The results showed that the GPR41/43/109A mRNA significantly decreased in the DSS group, whereas XSQ can increase their mRNA expression (Fig. 6H–J). These results suggest that XSQ exerts a therapeutic effect on colitis by regulating the production of SCFAs and promoting the expression of the GPR41/43/109A mRNA.

### XSQ suppressed activation of MAPK/ERK/JNK pathway

**Fig. 7:**
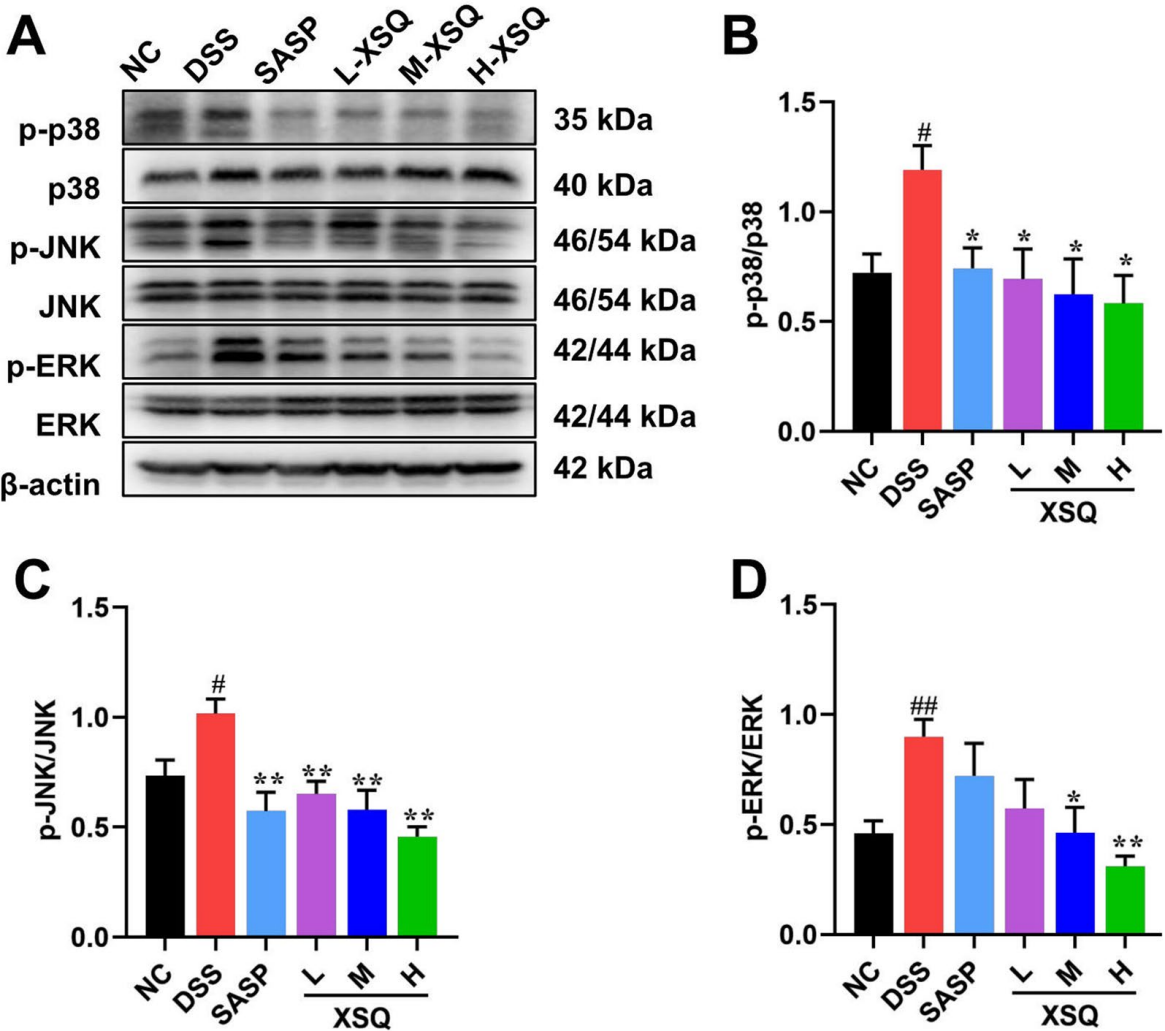
XSQ suppressed activation of MAPK pathway. **A** The representative images of western blotting of p-p38, p38, p-JNK, JNK, p-ERK, and ERK protein. **B**–**D** The expressional changes statistical results of p-P38/ P38, p-JNK/ JNK, p-ERK/ ERK protein by Image J software. Data were expressed as ± SEM (n = 3), ^##^*P* < 0.01 vs DSS group; *P < 0.05, **P < 0.01 vs Ctrl group

They were based on XSQ activating the GPR41/43/109A receptor and inhibiting the intracellular inflammatory response. Moreover, mitogen-activated protein kinase (MAPK) is a class of highly conserved signaling modules in eukaryotic cells. It is an essential member of the link between intra- and extracellular responses, mediating the transmission of extracellular signaling stimuli into the cell and regulating cellular processes such as growth, differentiation, migration, and inflammation [13]. In mammals, the family of MAPKs is divided into three main classes: extracellular signal-regulated kinases (ERK), c-Jun amino-terminal kinases (JNK), and p38 MAPK kinases. Various extracellular stimuli, including inflammatory cytokines, growth factors, cellular stress, etc., can activate the MAPK pathway. To explore how XSQ interacts with these pathways, we measure critical proteins in the MAPK/ERK/JNK pathway signaling. In the DSS group, we observed a significant increase in protein expression p-p38, p-JNK, and p-ERK compared to the Ctrl group. However, XSQ significantly inhibited the expression of p-P38, p-P38, and p-ERK protein (Fig. 7). The results showed that XSQ treatment inhibited MAPK/ERK/JNK pathway activation and alleviated colitis-related symptoms.

### XSQ regulates tight junction proteins to protect the epithelial barrier

**Fig. 8:**
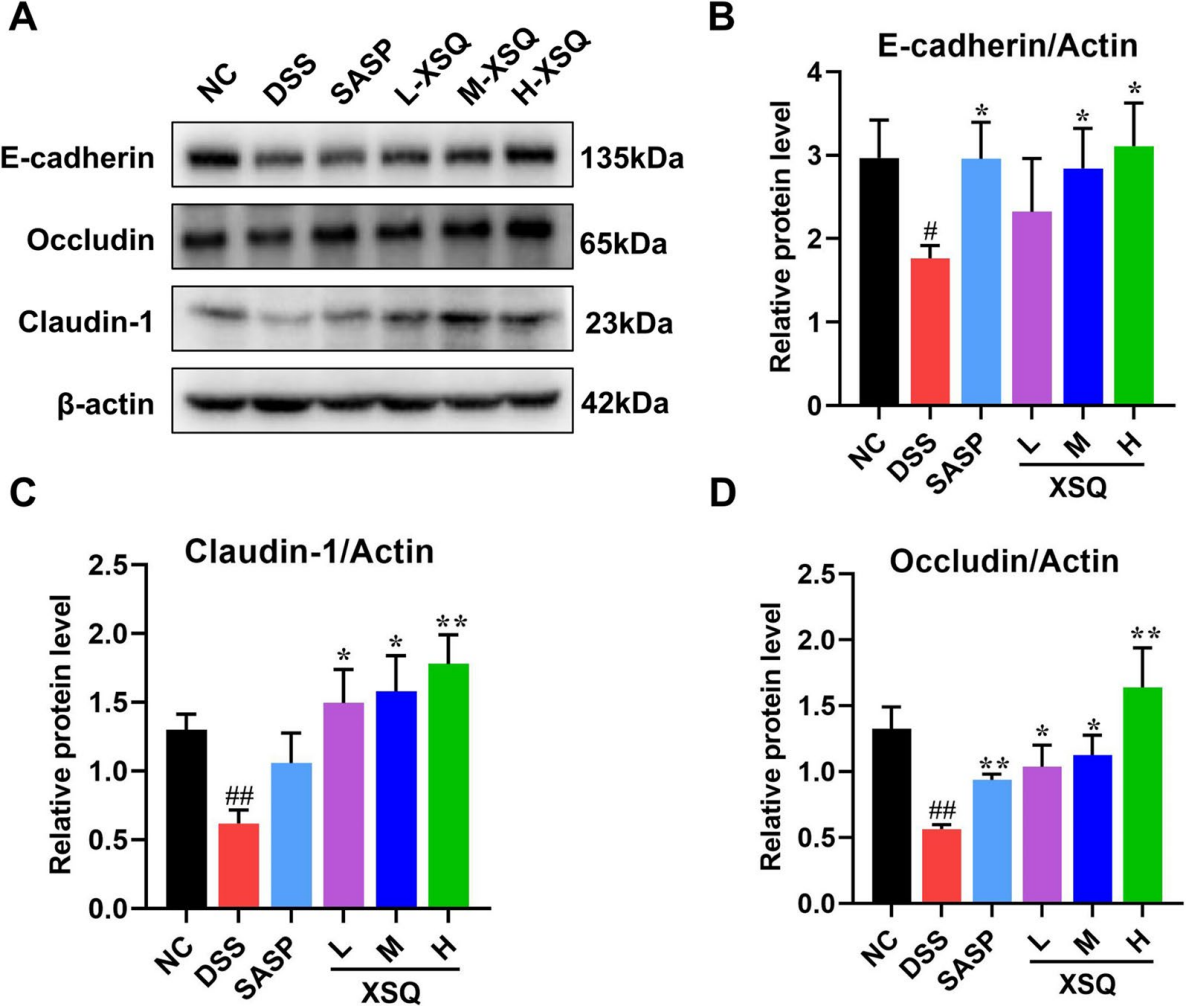
XSQ improved the gut barrier disruption in colon tissues. **A**–**D** The representative images of E-cadherin, Occludin, and Claudin-1 protein and the corresponding expression measured by image J software. Data were expressed as ± SEM (n = 3), ^##^*P* < 0.01 vs DSS group; *P < 0.05, **P < 0.01 vs Ctrl group

Tight junction proteins are a vital component of the epithelial barrier. The barrier filters, determining which ions and proteins can pass through. A disruption to the expression of the junction proteins would compromise the barrier’s integrity, allowing unwanted antigens to infiltrate the colon. In the DSS group, we observed significantly decreased Occludin, Claudin, and E-cadherin expression compared to the Ctrl group. This phenomenon was significantly reversed in the XSQ treatment group (Fig. 8A–D).

### Correlation analysis between physical and chemical indicators and intestinal microbiota

**Fig. 9:**
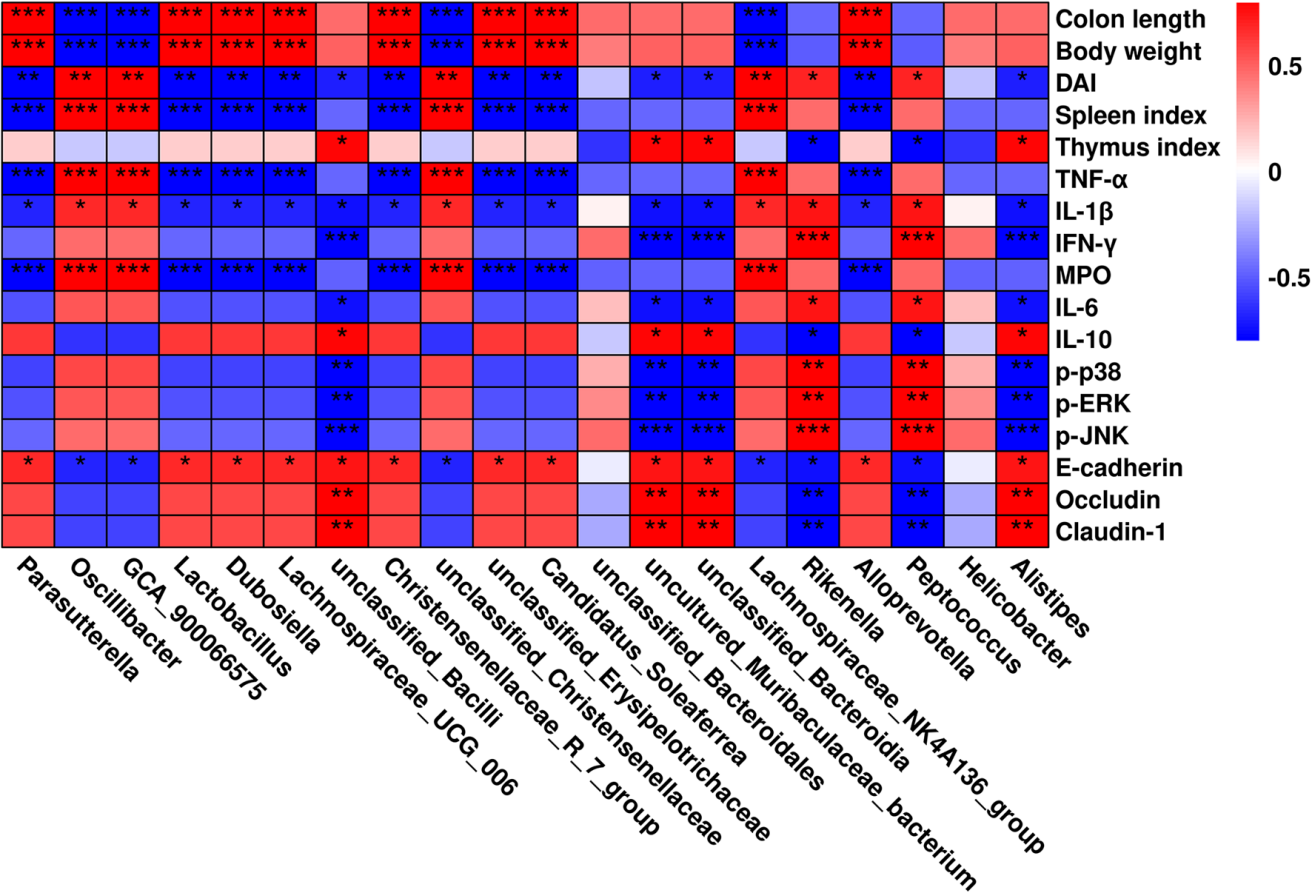
Correlation analysis of intestinal bacterial changes at genus level with colitis-related physicochemical indicators. *P < 0.05, **P < 0.01 and ***P < 0.001 vs Ctrl group

Spearman’s correlation analysis was performed to investigate the relationship between changes in the gut microbiota and related physicochemical indicators in mice with colitis. As shown in Fig. 9, the abundance of bacteria with the greatest change at the genus level was positive (*P* < 0.05 or *P* < 0.01) or negative (*P* < 0.05 or *P* < 0.01) in relation to the physicochemical indicators associated with colitis.

## Discussion

Colitis is an inflammatory bowel disease with rising global prevalence, characterized by symptoms such as abdominal pain, diarrhea, blood in stool, and weight loss [14–17]. This condition's economic and healthcare implications are substantial, necessitating effective treatment strategies. Current treatment regimens consist of administering 5-aminosalicylic acid drugs, corticosteroid injections, and long-term maintenance treatments. While temporary remission of conditions is possible, it is challenging to prevent a relapse. The persistent and refractory nature of colitis necessitates alternative effective treatment strategies.

Traditional Chinese Medicine (TCM), especially folk herbal recipes, has enormous untapped potential to serve as the basis for developing new drugs. Documented in the *Hubei Herbal Records*, minority groups in Enshi Tujia and Miao Autonomous Prefecture use XSQ in detoxification, anti-inflammatory, and analgesic recipes [18]. Modern pharmacological studies have expanded on these traditional recipes, and XSQ was found to have antibacterial, anti-inflammatory, antioxidant, and anti-atherosclerotic effects [19–24]. However, its specific application in inflammatory bowel diseases like colitis has been scarcely explored.

We found that the main active components in XSQ are quinic acid, gallic acid, and ferulic acid, among others, according to UPLC-Q-TOF–MS/MS analysis. Quinic and gallic acid are potent antioxidants that may help reduce oxidative stress in inflamed colon tissue damage [25, 26]. Ferulic acid, a known anti-inflammatory agent, may help modulate the gut microbiota and suppress the release of inflammatory cytokines such as TNF-α, IL-1β, and IL-6 [27, 28]. Current literature found the presence of (+)-Catechin-5-O-glcolitisoside, flavonoids, and epicatechins in XSQ, and they have potent antioxidant and anti-inflammatory properties and may support the growth of probiotics and strengthen the intestinal epithelial barrier [29, 30]. In addition, Zhu et al. found that epicatechin derivatives such as epicatechin gallate and epicatechin 3-O-(3-O-methyl) gallate may contribute to the overall anti-inflammatory and antioxidant effects of XSQ, possibly by inhibiting MAPK signaling and upregulating tight junction proteins such as Occludin and Claudin-1 [31].

The significant alleviation of pathological symptoms in DSS-induced colitis mice following XSQ treatment underscores its therapeutic potential. The dose-dependent reduction in the DAI and the improvement in colon length suggest that XSQ effectively mitigates the severity of colitis [32]. The attenuation of MPO activity and histopathological improvements [33], such as reduced inflammatory cell infiltration and enhanced colonic mucosal integrity [34], further support XSQ's anti-inflammatory and protective effects on the colonic mucosal. These findings collectively demonstrate that XSQ positively impacts the resolution of acute colitis symptoms and the restoration of typical colon structure and function.

The anti-inflammatory effects of XSQ are further highlighted by its ability to reverse the upregulation of pro-inflammatory cytokines (including TNF-α, IL-1β, and IL-6) and the downregulation of the anti-inflammatory cytokine IL-10 [35]. This cytokine modulation is essential for curtailing inflammation and promoting tissue repair [36].

The role of gut microbiota dysbiosis in the pathogenesis of ulcerative colitis is well-documented, with a decrease in beneficial bacteria and an increase in pathogenic bacteria as its main features [37, 38]. Our sequencing result revealed the distinct gut microbiota structure among the trial groups. XSQ treatment brings the microbial community closer to that of the healthy controls, which aligns with previous findings that DSS-induced leads to alterations in the gut microbiota of mice. The restoration of the population of *Firmicutes* and *Bacteroidota* following XSQ treatment suggests a beneficial shift towards a balanced gut microbiota; this shift may be integral to intestinal homeostasis.

XSQ can influence the levels of the downstream metabolism SCFAs by regulating gut microbiota. For example, Yang et al. and Yi et al. found that active components of XSQ enriched the growth of probiotics such as *Lactobacillus* and the *Christensenellaceae_R_7_group* [39, 40]. These probiotics are known for producing butyrate acid and acetate acid and are critical for maintaining the intestinal epithelial barrier [41, 42]. Our sequencing study reached a similar conclusion, and our cecal SCFAs assay result showed that XSQ treatment increased colon-friendly acids such as acetic acid, propionic acid, butyric acid, and valeric acid content. These short-chain fatty acids can activate G-protein-coupled receptors, such as GPR41/43/109A, on the membrane of intestinal epithelial cells and inhibit the activation of intracellular inflammatory pathway proteins and thus inhibit the inflammatory response, which helps to alleviate the symptoms of colitis. Our experimental results show that XSQ does increase GPR41/43/109A mRNA gene expression. The MAPK pathway is closely associated with the inflammatory response in colitis. In particular, the phosphorylation of MAPK proteins such as p-p38, p-ERK, and p-JNK exacerbates the inflammatory response. These proteins are heavily involved with the inflammatory cascade before the onset of colitis, so finding a solution to circumvent this molecular mechanism may negate inflammatory response in colitis patients. Our W.B. assay result showed that XSQ treatment suppressed the signaling of the MAPK pathway and showed potential for developing targeted therapy for colitis-related inflammatory response. In combination, XSQ treatment increased the relative abundance of pro-biotics and regulated their metabolites to mitigate DSS-induced colitis damage in mice.

Symptoms of colitis, such as diarrhea, blood in the stool, or colitis stem from a compromised intestinal epithelial barrier. Dysregulation of the intestinal epithelial barrier is a critical pathogenetic factor of colitis [43, 44]. Our western blot assay result showed that XSQ treatment could significantly upregulate the tight junction proteins such as Occludin, Claudin-1, and E-cadherin. These proteins are essential to the intestinal epithelial barrier and help regulate ions and particles passing through the barrier. Strengthening the barrier prevents infectious insults and other irritants from entering the colon and may help prevent the onset of colitis or aid in healing.

While our study provided exciting insights into our original hypothesis that XSQ promotes recovery from colitis, it has left a few questions unanswered. For example, the intervention phase of our trial was only 10 days. It is very short in comparison to the popular treatment regimens in practice. Therefore, we cannot explain the long-term outcome of XSQ treatment. In addition, one can argue that our sample size needs to be more significant to provide adequate statistical power in our results. While these are all valid causes for concern, we believe we have met all goals within the ambit of our original study design. We observed that XSQ treatment provided evident remission of symptoms in DSS-induced colitis in mice, and we observed improvement in multiple indirect metrics such as gut microbiota equilibrium, SCFAs content, cytokine, and protein expressions.

## Conclusion

In conclusion, our study demonstrated that XSQ could remodel gut microbiota imbalances and promote short-chain fatty acid production and the expression of GPR41/43/109A mRNA gene, regulate the secretion of inflammatory factors such as TNF-α, IL-1β, IFN-γ, IL-6, and IL-10, inhibit the activation of MAPK/ERK/JNK pathway, and increase the expression of tight junction proteins E-cadherin, Occludin, and Claudin-1, thereby restores the intestinal barrier function to alleviate the symptoms of colitis. Together, these properties suggest that XSQ could be an effective therapeutic agent for colitis and warrants further exploration for its application in treating inflammatory bowel disease.

## Method

### Materials and reagents

The plant raw material (XSQ) was collected (Enshi Tujia and Miao Autonomous Prefecture, Hubei, China) and identified by the laboratory of Dr. Dingrong Wan, School of Pharmaceutical Sciences, South Central Minzu University. Dextran sulfate sodium salt (DSS, MW: 36–50 kDa) and metaphosphoric acid were purchased from Aladdin Bio-Chem Technology Co., Ltd. (Shanghai, China). The Bicinchoninic Acid (BCA) protein assay kit and myeloperoxidase (MPO) assay kit were purchased from Beyotime Biotechnology (Shanghai, China). Mice tumor necrosis factor-α (TNF-α), interleukin-1β (IL-1β), and interferon-γ (IFN-γ) ELISA kits were purchased from NeoBioscience Technology Co., Ltd. (Shenzhen, China). Universal tissue fixative was purchased from Sevier Biotechnology Co., Ltd. (Wuhan, China). RNA extraction and PCR test kits were purchased from Nanjing Vazyme Biotech Co. (Nanjing, China). E-cadherin, Occludin, and Claudin-1 protein, P38 mitogen-activated protein kinase (P38), Phosphorylation (p)- P38, Jun N-terminal kinase (JNK), p-JNK, Extracellular regulated protein kinases (ERK), p- ERK and β-actin were purchased from ABclonal Technology Co., Ltd. (Wuhan, China). The cDNA transcription kit was purchased from ABclonal Technology (Wuhan, China).

### Preparation of XSQ and UPLC-Q-TOF–MS/MS analysis

The crude powder of XSQ was weighed and extracted twice, using a 6-times-volume of and 5-times-volume of pure water for 1 h each time. Then, the extract was filtered to obtain a filtrate. The filtrate was concentrated at relative density and then dried to obtain the extract powder for the sample.

The samples were analyzed using an Agilent ZORBAX RRHD Eclipse XDB-C18 column (2.1 × 100 mm, 1.8 μm) on the Waters H-Class ultra-high performance Liquid Chromatograph (Waters, USA). The mobile phase consisted of A (0.1% aqueous formic acid) and B (methanol) with the following elution gradient: 0–1 min, 5% to 18% B; 1–4 min, 18% to 24% B; 4–7 min, isocratic at 24% B; 7–12 min, 24% to 46% B; 12–15 min, 46% to 64% B; 15–19 min, 64% to 95% B. The column temperature was maintained at 30 °C, with a flow rate of 0.4 mL/min and an injection volume of 5 μL. Detection was performed at a wavelength of 280 nm.

Mass spectrometry was performed on AB Sciex Triple TOF 4600 high-resolution Mass spectrometer (SCIEX), and a heated electrospray ionization source (ESI) was used by negative ion detection mode. The sheath gas flow was set at 60 mL/min, and the auxiliary gas flow at 20 mL/min. The spray voltage was set to 3.5 kV. The capillary and auxiliary heater temperatures were both maintained at 380 °C. The scan mode was set to full M.S./dd-MS2, with full M.S. resolution at 70,000 and dd-MS2 resolution at 17,500. The scan range was m/z 100 to 1200, and the collision energy was 30 eV.

### Animal experiment

All experimental procedures were reviewed, approved, and supervised by the ethical board at South-central Minzu University (License No. SYXK (E) 2021-0089). All procedures were performed by the guidelines in the Guide for the Care and Use of Laboratory Animals [45].

C57BL/6 male mice (6–8 weeks old, 22 ± 2 g) were supplied by Liaoning Provincial Laboratory Animal Resource Center (License No. SCXK (Liao) 2020-0001). All mice underwent acclimatization with adequate food and sterile drinking water for 1 week before randomization.

On the 8th day, 42 mice were randomized into 6 groups (n = 7 each): control group (Ctrl), dextran sulfate sodium (DSS) group, positive group (SASP, 0.02 g/kg), low-dose XSQ group (L-XSQ, 0.04 g/kg), medium-dose XSQ group (M-XSQ, 0.08 g/kg), and high-dose XSQ group (H-XSQ, 0.16 g/kg). Interventions for each group were orally administered, all mice received assigned interventions, and mice from different groups were kept in different cages to avoid cross-contamination.

All groups other than the Ctrl group were provided a 3% DSS solution for 7 days. In addition, all groups simultaneously received allocated interventions for 10 days.

Data on body weight, stool consistency, and stool color were collected daily to calculate the disease activity index (DAI) [46]. After the intervention period, all mice were ether-euthanatized, and their experiment-related tissues were extracted. Immediately following euthanasia, their organs were weighed, and colon length was measured. 0.5 cm of distal colon tissue samples from each mouse were fixed for histological analysis. The remaining colon and other extracted tissue samples were stored at − 80 °C for subsequent analysis.

### Histological analysis

The colon tissues were fixed with 4% paraformaldehyde for at least 24 h. First, an appropriate amount of colonic tissue was taken, dehydrated, deparaffinized, and performed microtomy (4 µm). Next, the samples were then stained for H&E [47]. The pathological morphology of the colon tissues was examined and photographed with an optical microscope (Table 1).

**Table 1:** Anti-body used for western blot experiment

Premary	Company	Cat	Dilution
IL-6	abcam	ab9324	1:1000
IL-10	abcam	ab33471	1:1000
occludin-1	ABclonal	A2601	1:1000
E-cadherin	ABclonal	A20789	1:1000
claudin-1	ABclonal	A2196	1:800
p-P38	ABclonal	AP0526	1:1000
P38	ABclonal	A4771	1:1000
p-JNK	ABclonal	AP1337	1:1000
JNK	ABclonal	A4867	1:1000
p-ERK	ABclonal	AP0974	1:1000
ERK	ABclonal	A4782	1:1000
β-actin	ABclonal	AC026	1:200,000

The pathology score was calculated with the following formula: Pathological score = (Tissue structural integrity + Epithelial cell loss + Inflammatory cell infiltration + Degree of edema) / 4. The specific criteria of the scoring standards are provided in Table 2.

**Table 2:** Pathological scoring criteria

Score	Structural integrity	Goblet cells	Inflammatory cell infiltration	Oedema
0	Completely intact	No loss	No symptom	No symptom
1	Basically intact	Minor loss	Minor infiltration in outer layer	Minor oedema in outer layer
2	Distinguishable from surrounding tissue	Medium loss	Severe infiltration in outer layer	Severe oedema in outer layer
3	Hardly distinguishable from surrounding tissue	Severe loss	Infiltration in inner layer	Oedema in inner layer
4	Structure disappear	Complete loss	Infiltration expand to surrounding tissue	Oedema expand to surrounding tissue

### Myeloperoxidase (MPO) activity assay

First, prepare a 5% colon tissue homogenate with reagent 2. Then, 40 µL of reagent 3 was added to 360 µL of homogenate, mixed, and incubated in a 37 °C water bath for 15 min. 40 µL of Reagent 4 and 600 µL of dye were added to a new tube, followed by 40 µL of the above solution. Afterward, the reagents are mixed well and incubated in a 37 °C water bath for 30 min. After incubation, 10 µL Reagent 7 was added, mixed well, and incubated in a 60 °C water bath for 30 min. Finally, the O.D. value was measured at the excitation wavelength of 460 nm, and MPO activity was calculated with the following formula:

$${\text{MPO }}\left( {{\text{U}}/{\text{g colon tissue}}} \right) \, = \, \left( {{\text{OD}}_{{{\text{sample}}}} {-}{\text{ OD}}_{{{\text{control}}}} } \right) \, /{ 11}.{3 } \times {\text{sample weight }}\left( {\text{g}} \right).$$

### Cytokine assay

First, 10% colon tissue homogenates were prepared with phosphate buffer solution (PBS) and centrifuged with 12,000 g at 4 °C for 10 min; the supernatant was collected and stored in − 80 °C storage for subsequent analysis. Testing was completed according to the instructions provided by the kit vendor.

### 16s rRNA sequencing

The test kits and sequencing platform are purchased from Beijing Bemac Biotechnology Co., Ltd. (Beijing, China) in this experiment segment. First, total DNA was extracted with the TGuide S96 Magnetic Soil/Stool DNA Kit. Next, 50 ng of DNA sample was prepared for V3-V4 region amplification; the primers used are 338F (5′-ACTCCTACGGGAGGCAGCA-3′) and 806 R (5′-GGACTACHVGGGTWTCTAAT-3′). Samples of the PCR amplification experiment are purified with an OMEGA DNA kit. Finally, the samples were sequenced on the Illumina Novaseq 6000 platform.

The raw sequencing output (stitch, quality control, remove chimera) was cleaned to obtain the high-quality tags sequence. Then, the USEARCH tool (version 10.0, https://www.drive5.com/usearch) was used to cluster tag sequences with 97% or more agreement, and a 0.005% threshold was used to filter OTUs. Next, QIIME 2™ (National Institutes of Health, U.S.) assessed α and β diversity. Finally, the Metastats^®^ tool (Center of Bioinformatics and Computational Biology, University of Maryland) was used to analyze species abundance [48, 49].

### Detection of SCFAs in cecum contents

First, 100 mg cecum contents were suspended in 100 µL methanol, vortexed, and centrifuged with 12000 g at 4 °C for 10 min. 80 µL supernatant was collected and mixed well with 16 µL 25% metaphosphoric acid and let set overnight at 4 °C. Then, the supernatant was centrifuged with 12000 g at 4 °C for 10 min and collected for subsequent gas chromatography.

Use TRACE 1300 GC and TR-FAME GC columns to perform gas chromatography; the specification of the columns was 60 m × 0.25 mm ID × 0.25 µm. The temperature program we designed was: start at 75 °C, increase steadily by 2 °C/min until 180 °C, and maintain at this level for 2 min [50, 51].

### Western blotting (W.B.) protein assay

First, the enzymatic digestion method was used to extract protein, and protein concentration was measured with a BCA test kit [52]. Next, proteins were separated using the SDS-PAGE method and transferred to the PVDF membrane [53]. Blocked with 5% nonfat dry milk diluted in TBST buffer, antibody 1 was incubated at 4 °C overnight. The next day, the membrane was washed with TBST buffer, and the appropriate amount of diluted antibody 2 was incubated for 1 h at room temperature. Afterward, The membranes were washed 3 more times with TBST buffer and then blotted by ECL [54]. Finally, the results were analyzed using the Image J^®^ software (National Institutes of Health, U.S.).

### Statistical analysis

We used ANOVA to compare the difference between trial groups on the GraphPad Prism software (version 8.0.2, La Jolla, CA, USA). The test significance threshold was set to *P = 0.05 and **P = 0.01. All data was reported as mean ± standard error of the mean (SEM).

## Data Availability

Data will be made available on request.
